# The New York Institute of Stomatology

**Published:** 1900-09

**Authors:** 

**Affiliations:** The New York Institute of Stomatology


### Reports of Society Meetings.

### THE NEW YORK INSTITUTE OF STOMATOLOGY.

A REGULAR meeting of the Institute was held on Tuesday evening, April 3, 1900, at the office of Dr. George S. Allan, 51 West Thirty-seventh Street, New York, the President, Dr. E. A. Bogue, in the chair.

The minutes of the previous meeting were read and approved.

COMMUNICATIONS FROM OFFICERS.

*Dr. S. E. Davenport.*—It grieves me to be obliged to inform the Institute that our dear friend and member, Dr. George A. Maxfield, of Holyoke, Mass., whom we expected to enter largely into our programme this evening, is plunged into the deepest sorrow on account of the recent death of his only son, a lad about ten years of age. I think it would be fitting, Mr. President, for the members of this Institute to give an expression of their sympathy through our secretary to Dr. Maxfield. I move you, sir, that our secretary be instructed to communicate to Dr. Maxfield our sympathy for him in his bereavement.

Motion seconded and unanimously carried.

[The report from Dr. La Salle anent the International meeting at Paris simply covers the official notices already published, and is too late to be of value to those attending the International Dental Congress. It is therefore omitted.—Ed.]

COMMUNICATIONS ON THEORY AND PRACTICE.

*Dr. W. St. George Elliott.*—I wish to present to the Institute a new form of sterilizer invented by my son. The apparatus consists of a cylindrical receptacle in which the instruments to be sterilized are placed. The whole is then covered with a hood, after which the apparatus is filled with formaldehyde gas. I can recommend it on account of its simplicity and the ease and quickness with which the desired purpose can be accomplished.

The second appliance is a simple automatic cut-off for gas to be operated by an ordinary alarm clock in shutting off the gas from the vulcanizer. The alarm being set for the time desired to shut off the gas, the rotation of the alarm-winding key releases a weight on the end of an extension piece attached to the gas tap, thus cutting off the gas.

This instrument is a broach-holder which is capable of holding a broach at any angle with the handle. It can also be used as an odontometer.

*The President.*—I have received a little communication from our friend and fellow-member, Dr. Barnes, of Cleveland. He has requested me to present to the Institute, with his compliments, an amalgam crown, together with a little paper descriptive of it.

DR. BARNES'S COMMUNICATION.

Dear Doctor,—I have a new thing in the shape of an amalgam crown which I will send on. If you think it of sufficient merit, present it to the Institute.

Briefly, it is this: A sectional plaster model is taken of the crown of a natural molar tooth. Different models will give a selection of crowns, and they can be made in the laboratory by the assistant, and kept on hand for use at any time.

Quick-setting amalgam is then burnished to the sides of the model, leaving the centre hollow and a hole through the centre of the crown. When the amalgam has set it is polished, and you have a hollow amalgam crown which may be set upon a badly decayed root with quick-setting amalgam.

The root is prepared as for any large filling, with pins extending into the coronal portion; a crown is selected of the correct size and shape; a narrow band of phosphor bronze is placed about the cervical portion of the root, extending up to hold the crown in position. Prepare amalgam rather soft and pack into the root portion; the crown is then forced into position, being held in place by the band. The hole in the top of the crown permits of manipulation from above with instruments. When on a few minutes, the band may be removed and the excess of amalgam about the cervical portion removed in the usual manner. Those who have seen the crown here are quite well pleased with it.

Fraternally yours,

Henry Barnes.

Dr. Barnes also called attention to a little clamp which he has found extremely useful and which is described in the March number of the *Dental Cosmos,* page 258.

*Dr. Davenport.*—I should like to say that the crown, upon examination, seems worthy of highest commendation wherever an amalgam crown could be used.

*Dr. S. H. McNaughton.*—I think it would be better if, instead of making the band of bronze, he would make it of platinum or gold and leave it in place permanently.

*Dr. G. E. Adams.*—With your kind permission, I would like to present to the Institute models of a case which was of great interest to me. It is the case of a boy fourteen years of age, who has no lower permanent teeth and but four teeth in the superior maxilla.

The boy was brought to my office by Dr. Watson, who prepared these models. His temporary teeth were extracted and no permanent teeth erupted. I do not know that it is new to most of the gentlemen present, but it is the first case of the kind which I have ever seen.

*The President.*—Such cases as this are not unknown, but we are greatly obliged to Dr. Adams for presenting the models to the Institute.

The paper of the evening, "Some Methods of extirpating Pulps, and Subsequent Treatment," by Dr. J. B. Locherty, was read.

(For Dr. Locherty's paper, see page 590.)

### DISCUSSION.

The following letters were read by Dr. Davenport:

From Dr. E. C. Briggs, of Boston:

It is not my intention to present a finished article to the Institute of Stomatology, but only to jot down a few of my theories and practices in relation to the subject-matter of Dr. Locherty's paper. I strongly advocate the surgical removal of the pulp, because I feel that when it is successfully done the tooth is in no sense devitalized. In my opinion, the fully matured tooth is not dependent upon the pulp, while, on the other hand, the presence of the pulp is often a menace and cause of serious trouble,—as witness the nervous disturbances resulting from pulp-stones and exostoses,—and seriously complicating the treatment of pyorrhœa alveolaris; and if the pulp is removed surgically and aseptically, the tooth will be stronger and more serviceable than before. When surgically removed there is no injury to the pericementum or to the nerves running from the root-canals to the pericementum. In other words, the tooth's vital connection with the body is not disturbed; one has only removed the constructive organ, and, construction being complete, it becomes a superfluous tissue. Of course, to make a perfect result, asepsis and complete mechanical stopping of the canals must supplement the removal. The fact that there are failures to do one or both of these things, owing to faulty manipulations or malformations, does not militate against the underlying principle in the case.

On the other hand, it is my belief that the destruction of the pulp with arsenous acid is too often fraught with danger far-reaching in its results. The tooth too often becomes literally a dead tooth, and too often the tissues beyond the tooth are affected, becoming the starting-point of abscesses, dentigerous cysts, and the like.

While I do not believe that arsenic is directly the cause of death, I do think that many cases, fatal and serious, may be due to actinomycosis, which finds its nidus in the necrotic tissue caused by arsenous acid.

But "necessity knows no law," and I, too, use arsenous acid when it seems necessary. It is my aim, however, in all cases to use it only in part, trying to get the pulp from the canals by surgical means before the arsenic has reached that point. Arsenic is a deadly and subtle poison, and the term caustic or escharotic is no description of its insidious and penetrating toxic action.

It was in January, 1892, that it first occurred to me to use cocaine in the surgical removal of the pulp, and I wish that all might share with me the great satisfaction this method gives. I use a twenty per cent. aqueous solution of the hydrochlorate, and with blunt-pointed syringe or plugs of unvulcanized rubber endeavor to throw the solution *around* the pulp, not into it. It is my theory that the cocaine solution jackets the pulp, since in all cases when, from too sharp or too small-pointed a syringe, the pulp has been penetrated, the result has been unsatisfactory.

The injection is begun slowly, so that the first pressure against the pulp shall not produce a shock; then, as the solution gradually forces itself around the pulp, the pressure becomes harder, until finally the piston is pressed down as hard as may be. If all has gone well, there has been no pain, and immediately one can bur out the pulp-chamber and with barbed broaches remove the pulp from the root-canals. The roots are then to be dried out and filled immediately. Waiting even half an hour, as I have done where an emergency case has had to be "sandwiched" in, will show that all life has not been taken from the tooth, as the passage of a broach into the root gives as much pain as if the pulp were still there.

The cases of surgical removal of the pulp by means of cocaine done in my office by myself and my four associates number in the hundreds, and the testimony of all is that it is one of the greatest comforts of modern dentistry.

I will not weary you with citation of cases, but state that they include the patients who come to you with toothache from exposed pulp, whether from disease or fracture, neuralgia from pulp-stones, and the rare but necessary instances where teeth must be used as supports for bridges. Some cases of single-rooted teeth have been dismissed from the chair with the root filled within a half-hour from beginning the operation. There are some cases of failure, a small per cent., however, of the whole number treated. In these cases the cocaine has little or no effect. Why, I do not know. It may be non-susceptibility to the drug, or the pulp may be adherent to the walls, thereby not allowing the cocaine to get in around it.

The theories and observations contained in this paper are based entirely on clinical experience, and I am aware that the more scientific of you may pick flaws in it.

From Dr. H. W. Gillett, of Newport, R. I.:

Dating from the reading of a paper by Dr. E. C. Briggs before the Harvard Odontological Society, in about 1890, in which he advocated pulp extirpation with the aid of cocaine, I began following that procedure as regular routine practice, at first for selected cases, but soon for practically all cases.

For at least six or seven years, more than ninety-nine per cent. of all my pulp removals have been by that method, generally using the cocaine with a syringe, as Dr. Briggs does.

It is my habit to pump up ninety-five per cent. carbolic acid after removing the pulp, using fine jewellers' broaches or Donaldson cleansers, and, unless prevented by hemorrhage, to fill the canals at the same sitting. In my hands this procedure has resulted in the retention of good color in the pulpless teeth in all cases, and has been equally satisfactory in leading to practically universal good results as regards usefulness and comfort of the teeth so treated.

So far as I am able to trace them, the unsatisfactory results in several years of this practice are limited to one case of failure to get the tip of a lower molar pulp and consequent persistence of sensitiveness, and one case of abscess due to the disappearance of the salol root-filling in a tooth so treated.

It frequently happens that there is more or less soreness for a few days after the operation, this being often dependent (apparently) upon a tendency to go too far in the effort to make sure of all the pulp, or sometimes to forcing the cocaine solution too hard.

In my hands I have not found the procedure productive of more discomfort at the time in the average case than the older arsenic method, often the reverse, and I have found it so much more certain to result in a permanently useful and satisfactory-appearing tooth that I regard it as an essential to the satisfactory practice of our profession. The practical certainty of freedom from objectionable discoloration with this process cannot be too strongly brought out, and I feel that it should be insisted upon as the only admissible method of practice for pulp extirpation in, at least, the ten anterior teeth.

For molars there may be room for difference of opinion, but as regards my own practice I adhere to it there also, in spite of the greater difficulties (with very rare exceptions), because of the better average condition of the teeth as compared with those in which arsenic has been used.

From Dr. B. Holly Smith, of Baltimore:

My views and my practice have been so fully set forth in the paper read before the National Association in 1888, in Omaha, also in the paper read before the Northeastern Dental Association at Holyoke, Mass., in the fall of 1899, that I feel that it would be only a multiplication of words to enter into a discussion of this subject, except to say that continued experience in doing away with the dental pulp, convinces me that, wherever it can be done, the surgical course should be pursued, using cocaine as the obtundent. Such a very large proportion of pulpless teeth in which arsenic has been used as the devitalizing agent have given trouble, as compared with the very small proportion of teeth made pulpless by the surgical method, that this is sufficient proof of the superiority of the latter method. I still adhere to the plan of electrical osmosis, or producing the first anæsthesia incident to the exposure with cocaine cataphorically applied.

I should like very much to be at the meeting, but my college work, together with the fact that I have already been out of the city a great deal, compels me to remain at home.

*Dr. Adams.*—The paper of the evening has been of great interest to me, as have the communications read by Dr. Davenport. In the practice of the surgical extirpation of the pulp my experience is quite limited. Unfortunately I seem to have met with a large number of patients who are very susceptible to the effects of cocaine, and for that reason I have not used it to any extent. My method has been to make a paste of arsenic, morphia, and menthol, which is sealed into the cavity with cement to prevent leakage. I find this effective, and have never seen ill effects from discoloration.

*Dr. I. Franklin Wardwell.*—My experience of late in devitalization has been very limited, indeed, and unfortunately being detained this evening, I did not hear the paper, consequently I am not prepared to discuss it. I have no doubt that for the anterior teeth the use of the cocaine for extirpation would work very satisfactorily. In this connection I am reminded of a preparation given me some years ago by my friend Dr. Forberg while I was in Stockholm. It was a peculiarly prepared cotton for filling root-canals, called "kopvadd," and when so prepared cannot be ignited. I remember filling the canals of a tooth with this preparation with success, the tooth, I was told, having a history of nineteen previous treatments and fillings, and each time the filling had to be removed.

*Dr. E. H. Babcock.*—I have been very much interested in the paper, although I am one of those who have used arsenous acid preparations almost exclusively. Regarding the dangers of injecting cocaine into a tooth, it has seemed to me that they would be very slight, as there would be very little chance for absorption through the apical foramen. The essayist spoke of using the aromatic sulphuric acid in cleansing pulp-canals. I would like to ask, Why use the aromatic in preference to the plain sulphuric acid? As I understand it, the action of the sulphuric acid is simply to dissolve the inorganic part of the tooth.

Regarding the matter of water in the tubuli for the more ready distribution of whatever medicament is used, my plan has always been to remove all fluids from the canal, thus allowing capillary action to carry the antiseptics thoroughly into the dentinal substance. As to my own method, I do not claim any pre-eminence of success, but I can say that it has given my patients and myself great satisfaction. I have always used arsenous acid combined with sulphate of morphia, oil of cloves, and sufficient creosote to make a paste. When I desire to devitalize a tooth, I take a piece of cotton about the size of the head of a pin, and after dipping it into ninety-five per cent. carbolic, I place upon it a few crystals of cocaine, which are readily dissolved in the carbolic. I then dip this into the paste and apply. The anæsthetic effect of the cocaine, lasting from three to five minutes, is supplemented by the action of the carbolic acid, and that, in turn, by the morphia. It has been my experience that the patient has no pain after the first few minutes. The only condition where I have trouble is where the pulp is partially dead. This is probably due to two causes: first, the congested circulation, which prevents absorption, and, secondly, that there is considerable devitalized pulp to penetrate before the medicament can reach the vital portion. These, I believe, are cases which would be particularly adapted for the use of cocaine. I do not believe in using a very large quantity of arsenic, nor in leaving it in longer than twenty-four hours (as a rule). After the first application of arsenic any remaining vitality can be overcome by the use of carbolic and the crystals of cocaine.

Regarding the matter of discoloration, I have never considered that the method of devitalization by the dentist had anything to do with it. It is a matter dependent entirely upon proper cleansing and filling of the tooth.

*Dr. J. H. Downes.*—I have listened to everything which has been said this evening with a great deal of interest, particularly regarding the destruction of the pulp. So far as my method is concerned, I use arsenic altogether. I never have tried the surgical operation, but, as I have never taken particular pains to protect the pulp from moisture, I probably should not have been successful. I have found that arsenic applied in small quantities successfully destroys the pulp with very little inconvenience to the patient, and I doubt very much if the surgical method could be accomplished without some pain. I think, myself, that I prefer the arsenic. Regarding discoloration, I believe that is independent of the method of devitalization, as it seems to occur in all dead teeth irrespective of the cause. There must be many cases where it would be impossible to perform the surgical extirpation, and again the long time taken for the operation should be taken into consideration.

*Dr. C. O. Kimball.*—I have listened to the paper with a great deal of interest, and as it is directly in line of my own practice, I may be permitted to say a few words on one or two points.

In the first place, my observations convince me that a tooth destroyed by arsenous acid does not discolor as much as a tooth which dies a natural death. As I look back over my cases this seems to be the invariable observation. My practice has, for a great many years, been to use arsenous acid for devitalization, but about the time Dr. Briggs spoke of using cocaine, eight or ten years ago, I began using it in my practice, not so much for aid in devitalizing the pulp as for obtunding the gum in the use of little retaining wedges. I now use it continually, and while at first I had a certain number of disagreeable cases, I have lately had almost none. I

will say this about the use of cocaine, that if used in the mouth in small quantities, not more than one-half to one minim of a two per cent. solution, being careful not to let the patient swallow any, it can be used without any difficulty whatever. I use it in all sorts of patients. I have recently been using eucaine, but I have found a larger number of cases followed by infection in the use of eucaine than with the use of cocaine. I am led to wonder if this is not due to the different action of the two drugs on the capillaries, eucaine relaxing, while cocaine has the opposite effect of contracting them.

As to the extraction of the pulp under cocaine, I can speak positively about that, as I have used it many times. For the anterior teeth, where there is a single root, it seems to me that it is far better than any other method. There is no question in my mind but that it can be done with almost no pain. Indeed, I have tried it many times with this result, questioning the patient very carefully in all cases. In cases difficult of access I prefer the arsenous acid method.

*Dr. E. H. Raymond.*—The paper is one of interest, and I regard it as a valuable contribution. In 1885 I made some extended experiments with cocaine, injecting the drug hypodermically into the inferior dental and mental foramina, for the lower teeth, and into the infra-orbital to anæsthetize the anterior superior teeth. It was found that good anæsthetic effect was produced in this way in some cases, but results were not uniformly successful, as in some temperaments it acted as a diffusive stimulant without producing anæsthetic effect.

Not being able to get uniform results with it for anæsthetizing and extirpating pulps, I abandoned its use. I have lately been using eucaine, as it seems to be more readily absorbed by the pulp-tissue.

As to arsenous acid, I think the trouble is, generally, that too much of the drug is used. A very minute quantity is all that is necessary to devitalize the pulp in any tooth, although the application may have to be repeated. Great care should always be used to seal it in the cavity tightly, as unfortunate results might follow if it comes in contact with the surrounding tissues.

When a pulp is found to be exposed in a cavity running some distance under the gum, and has become congested, what is the best means of making the application of arsenous acid to destroy it, and how would the surrounding tissues be protected?

*Dr. A. R. Starr.*—I can only say that I have had very little experience in the use of cocaine for the extirpation of the pulp. The only cases where I have used it have been in the anterior teeth, and here sometimes I have been successful and sometimes not. The great difficulty is the length of time required. It is unfortunate that our patients will come to us in a condition requiring extirpation of a pulp at times when we are very busy, and when it would be inconvenient to perform the operation under cocaine, although we can generally find time to apply some arsenic.

In the case just spoken of I have had the best success by using a little temporary stopping, first filling the cervical portion of the cavity with this and leaving the point of exposure uncovered. Then apply the arsenical preparation under a metal cap, and fill the remainder of the cavity with temporary stopping.

*Dr. B. F. Luckey.*—It does not seem to me necessary to go very deeply into this subject. The subject of arsenic, its use and abuse, is so old, it seems almost elementary. Regarding the use of cocaine and eucaine in the extirpation of pulps, after a careful and conscientious use of these drugs I have gone back to my first love, arsenic. What I am unable to accomplish with arsenic I cannot accomplish with cocaine, except for rapid extirpation, and here I find the chloride of ethyl serves my purpose better. By covering the tooth with bibulous paper, and by using an alternating current, I might say, of the ethyl chloride, I am able to chill the tooth in such a way as to render the extraction of the pulp almost painless.

Where the cavity is so far under the gum as to render it difficult to apply the arsenic, my practice has been to drill in from the crown and devitalize the tooth in this manner. It is not necessary to go too far the first time. Drill as far as possible and apply the arsenic for a day or two, and then drill a little farther. In this manner it can be accomplished without trouble. The idea that it is absolutely necessary to place the arsenic in the cavity of decay is a mistake. I have fresh in my mind a case of a suit for damages for five thousand dollars. The cavity was on the labial surface of an upper cuspid tooth extending under the gum. Arsenic was used in a very slovenly manner, with a covering of cotton and sandarac varnish. As a result the patient not only lost that tooth, but the first bicuspid as well, and a large portion of the superior maxilla, from necrosis. If that dentist had either followed Dr. Starr's method or had drilled into the tooth from another point, he would have saved his patient and his money.

*Dr. De Witt L. Parker.*—I would like to say that my brother and I have had some success in the use of compressed air for immediate extirpation of the pulp.

*Dr. E. S. Robinson.*—My method of procedure is generally the use of arsenous acid, though I have several times painlessly extirpated the pulp by means of cocaine. I have done this in teeth that have been broken off. I injected the cocaine by means of a hypodermic syringe, with a bent needle, but did not use the rubber dam. In speaking of discoloration, I think a great many times the discoloration is the result of the medicaments used in subsequent treatment. I find that oil of cinnamon causes a yellowish color.

*Dr. J. G. Palmer.*—One of our associate members, Dr. J. A. Libbey, of Pittsburg, Pa., suggested, in writing to me on this subject, the use of formaldehyde with cocaine. He claimed that it materially hastened anæsthesia. I have used it in only one instance, when I desired to remove the pulp at once. A boy about eleven years old was brought to me with a fractured right superior central, the pulp protruding. The accident had occurred twenty-four hours before, and, as we all know, the child was suffering a great deal of pain. I put on the rubber dam, and applied cocaine and formaldehyde in small quantities, removing the pulp in less than half an hour absolutely without pain.

Referring to cases when a buccal cavity extending under the gum presents, with an exposure of the pulp, as spoken of by Dr. Raymond, it would seem wiser perhaps to combine the two methods instead of immediately applying arsenic and risking the possible sloughing of the gum-tissue. By the use of cocaine, a sufficient quantity of the pulp-tissue can be removed to permit placing the arsenical application within the pulp-chamber, where it can be sealed.

*Dr. L. C. Leroy.*—I have been very much interested in the paper, especially in that portion touching upon the toxic effects of the arsenic being allayed by the use of the perchloride of iron, having had two or three troublesome cases. In one, occurring in the mouth of a brother dentist, where extreme caution had been exercised, it was impossible for the drug to have exuded except through the tubuli, possibly where the dentine, cementum, and enamel approximate, and this I feel convinced it did. The disturbance was allayed by the use of the perchloride of iron. In another case, inflammation became quite extensive, even extending to the next tooth. Perchloride of iron was injected hypodermically. The change in twenty-four hours was remarkable.

Dr. Locherty's paper is of especial value because he outlines treatment for such conditions, which is the first instance to my knowledge such has been done. I gained my information for the treatment of arsenical poisoning from a medical book treating of poisons and their antidotes, after having searched in vain through dental text-books and a host of dental periodical literature.

As Dr. Starr has said, one objection to the cocaine method for devitalizing pulps is that patients requiring such treatment come to us at an inconvenient time. The use of the arsenous acid gives us almost universally good results. I have yet to record a case where I have lost a tooth by its use. In the use of cocaine, what becomes of the vital tissue which must necessarily be left in the canal? Does it retain its vitality, or does it subsequently become devitalized? As to the hemorrhage after the use of cocaine, this is controlled very easily by the use of the peroxide of hydrogen.

*Dr. F. Milton Smith.*—After listening to the paper, I congratulated myself that I had learned the secret of successful devitalization of the pulp, and was going home to-morrow to try it on a young man. Then I listened to Dr. Downes, who told us that if we take arsenic and apply it in the right way we will not have any trouble. I have been using arsenic ever since a professor in operative dentistry told me a number of years ago that I must never use it under any condition. I have not found it entirely satisfactory, as so many gentlemen have. I have heard a few gentlemen speak to-night of having no pain following its use. Unfortunately many patients in my vicinity have pulps which do not respond favorably to the application of arsenic, and I have lost teeth from the excessive pain caused by it. I find I am the only one who ever has this difficulty. However, I am inclined to try Dr. Locherty's plan, as I have failed so badly with my own for the last twenty-five years. I remember some months ago having heard the President of the Institute make some remarks in reference to the difficulty of removing the pulps from hair-like canals in molars. The question comes to my mind whether Dr. Locherty is able to remove these pulps in all cases. Dr. Wardwell has suggested the use of kopvadd. I am very glad to know that we have such an agent, but am inclined to think that Wardwell had as much to do with the cure of that case as the kopvadd. A lady came into my office to-day with a lateral incisor which has been treated for a year and a half. I shall treat the same with a little Smith, some carbolic acid, iodoform, and gutta-percha, and hope in less than a week to see it well.

*Dr. Locherty.*—I have been very much interested in the remarks of the different gentlemen present in regard to the treatment of the pulp, for and against arsenic, and it bears out my statement that there is a great·diversity of opinion in this regard, and probably will be for some time to come. I used arsenic for a number of years, and then determined to try the cocaine method. I used it with varied success for a time and then went back to the arsenous acid. I then had a patient who was taken sick and unable to return to the office at the appointed time, and for that reason I abandoned the use of it altogether and commenced the devitalization and extirpation of pulps by the use of cocaine. I have continued to use it ever since, and find that I can accomplish just as thorough and more satisfactory results. So far as pain is concerned, the operation can be performed painlessly, provided sufficient time be taken. I will admit that it does take time, but the operation is such an important one that I believe the time spent is just as important and should be paid for the same as time spent in filling with gold, or any other skilful operation. I have found that cocaine has been more effective than eucaine, although my experience with the latter might have been too limited. Ethyl chloride I have never used. It seems to me that cold applied to the root of a tooth might cause sloughing if applied too long. As regards the preparation of iron to use to counteract the arsenic, the oxide is the best according to chemistry. To check a hemorrhage when extirpating a pulp under cocaine, peroxide of hydrogen is very effective. As cocaine has the characteristic of contracting the capillaries, there is very little hemorrhage generally in using such a method, for instance, as described in the typical cases in the paper.

*The President.*—On this subject, "Some Methods of extirpating Pulps and Subsequent Treatment," it seems that we have heard of various methods. There are a few points that it might be well to recall. One is the question asked by Dr. Raymond regarding the use of arsenic in a cavity that was partially covered by gum. A few years ago Dr. Thomas, of Madrid, mixed cotton with arsenic, cloves, morphia, and oil of cinnamon, cut his cotton up in sufficiently fine pieces that a little bit could be taken and put anywhere and covered with gutta-percha and wax. This cannot ooze. It is one way of accomplishing Dr. Raymond's purpose, and I find it very satisfactory.

In all of the papers brought before us this evening, I have had a feeling that the gentlemen presenting them were not careful enough to discriminate. There are thirty-two teeth in the mouth with as many different characteristics as there are teeth. While it might be very easy to remove the pulp in the manner referred to in the six front teeth, it seems to me that it would be very difficult to remove it in this manner in some of the molar roots. I am sorry that Dr. Locherty did not bring that before us a little more clearly. I think I may venture to say that the fact that extreme heat and extreme cold are both anæsthetic was not brought out as sharply as it might have been. I have taken out pulp after pulp by this method; this in the six front teeth. The most notable one was the case of a gentleman who was not in the chair over twenty minutes. I enveloped the tooth in cotton and threw chloride of ethyl upon it; then removed the pulp and filled the root cavity. I remember the case of a little patient I had in the chair with an exposed pulp in a central incisor. I told him to hold on tightly, as I was going to hurt him badly. I had a smooth broach, the point of which had been turned at right angles, making a hook. I dipped this in creosote only and passed it up gently along the side of the pulp. When I had passed it up as far as I thought it ought to go, I rotated it gently with my fingers, and out came the pulp. It did not hurt much. The inference to be drawn from this is that the healthy, living pulp is not such a very sensitive thing.

When cataphoresis came along, we very soon were told that pulps were of little value; that they had performed their function in the adult tooth, and could be extracted with ease and without injurious effects. Strange as it may seem, a crown-and-bridge man one evening at the Odontological was the only man to champion the unfortunate tooth-pulp. The question here arises, When is the function of the pulp ended? I remember the case of a gentleman who had worn his teeth to the gums by abrasion. A set of teeth was made for him covering these teeth. I do not know when the function of the pulp ended in this case, as secondary dentine was formed as fast as the abrasion took place. This gentleman was about sixty years of age.

Regarding the length of time arsenic can be left in a cavity, I think it has been stated by Dr. Westcott that he has left a treatment of arsenic in for several months without trouble, and it has been said to have been left in as long as three years. I think the fact has been pretty well established that as long as any arsenic remains there is preservation from putrefactive decomposition. Arsenical preparations are used in the preservation of the skins of beasts and birds. The gentleman whose patient returned with the treatment of long standing stated that he found the pulp in a "browned" condition.

I wanted, in connection with the surgical removal of the pulp, to call the attention of the reader of the paper to the many peculiarities in the shapes and sizes of the pulps, such as the lily-shaped canals. My son, Dr. Frederick Bogue, had occasion, the other day, to examine a bicuspid tooth in which a gentleman on the other side of the water had, as he supposed, extracted the pulp and filled the canal. The patient came in with an abscess. Upon removing the filling, a small hole was found where the pulp ought to be, with plenty of pulp on either side. As a result, the tooth had badly discolored. I think these are cases which the gentlemen advocating the surgical removal of the pulp do not always bear in mind.

*Dr. Locherty.*—Probably, in the cases mentioned by the President, it would be impossible to get out all the pulp by any method. We can only do our best.

Adjourned.

FRED. L. BOGUE, M.D., D.D.S.,
*Editor The New York Institute of Stomatology.*

